# Gamified physical activation of young men – a Multidisciplinary Population-Based Randomized Controlled Trial (MOPO study)

**DOI:** 10.1186/1471-2458-13-32

**Published:** 2013-01-14

**Authors:** Riikka Ahola, Riitta Pyky, Timo Jämsä, Matti Mäntysaari, Heli Koskimäki, Tiina M Ikäheimo, Maija-Leena Huotari, Juha Röning, Hannu I Heikkinen, Raija Korpelainen

**Affiliations:** 1 Department of Medical Technology, University of Oulu, Oulu, Finland; 2 Aeromedical Centre and Diving Medical Centre, Centre for Military Medicine, Finnish Defence Forces, Helsinki, Finland; 3 Institute of Health Sciences, University of Oulu, Oulu, Finland; 4 Department of Sports and Exercise Medicine, Oulu Deaconess Institute, Oulu, Finland; 5 Department of Computer Science and Engineering, University of Oulu, Oulu, Finland; 6 Center for Environmental and Respiratory Health Research, University of Oulu, Oulu, Finland; 7 Faculty of Humanities, Information Studies, University of Oulu, Oulu, Finland; 8 Faculty of Humanities, Cultural Anthropology, University of Oulu, Oulu, Finland; 9 University Hospital, Oulu, Finland

**Keywords:** Internet, Social network, Transtheoretical model, Physical activity, Obesity, Sedentary behavior, Tailoring, Effectiveness, Behavior change, Gamification

## Abstract

**Background:**

Inactive and unhealthy lifestyles are common among adolescent men. The planned intervention examines the effectiveness of an interactive, gamified activation method, based on tailored health information, peer networks and participation, on physical activity, health and wellbeing in young men. We hypothesize that following the intervention the physical activation group will have an improved physical activity, as well as self-determined and measured health compared with the controls.

**Methods/design:**

Conscription-aged men (18 years) attending compulsory annual call-ups for military service in the city of Oulu in Finland (n = 1500) will be randomized to a 6-months intervention (n = 640) or a control group (n = 640) during the fall 2013. A questionnaire on health, health behaviour, diet and wellbeing is administered in the beginning and end of the intervention. In addition, anthropometric measures (height, weight and waist circumference), body composition, grip strength, heart rate variability and aerobic fitness will be measured. The activation group utilizes an online gamified activation method in combination with communal youth services, objective physical activity measurement, social networking, tailored health information and exercise programs according to baseline activity level and the readiness of changes of each individual. Daily physical activity of the participants is monitored in both the activation and control groups. The activation service rewards improvements in physical activity or reductions in sedentary behaviour. The performance and completion of the military service of the participants will also be followed.

**Discussion:**

The study will provide new information of physical activity, health and health behaviour of young men. Furthermore, a novel model including methods for increasing physical activity among young people is developed and its effects tested through an intervention. This unique gamified service for activating young men can provide a translational model for community use. It can also be utilized as such or tailored to other selected populations or age groups.

**Trial registration:**

ClinicalTrials.gov Identifier: NCT01376986

## Background

Particularly in industrialised wealthy societies physical inactivity causes 1.9 million deaths per year [1] because of its several adverse health effects [2]. Physical activity provides important health benefits already in adolescence, including reduced symptoms of depression and anxiety, improved physical fitness, as well as reduced body fatness and favourable cardiovascular and metabolic disease risk profiles [3]. Positive health effects of a physically active lifestyle among youth are tracked to adulthood, such as lesser amount of adult obesity [4,5]. Sedentary behaviour and physical activity have also been shown to persist and track from youth into adulthood [5,6]. Despite the known benefits of physical activity on health and future life opportunities, recent evidence consistently demonstrates that a majority of adolescents do not meet current physical activity and public health recommendations of at least 60 minutes per day of moderate or vigorous intensity activity on at least five days per week [3,7,8]. In fact, even less than 10% of the 16–19 years old meet these recommendations [9]. Obesity has become more common while physical fitness has deteriorated markedly. The change in weight has been rapid where the average weight of male adolescents has increased by more than six kilos over a period of 13 years [10]. At the same time, physical performance has declined dramatically [11,12].

Recent evidence underlines the importance of focusing on the balance of light-intensity activities and sedentary behaviours due to the deleterious effects of sitting on health and wellbeing [13]. A meta-analysis showed a dose–response relation between increased sedentary behaviour and unfavourable health outcomes in children and youth [14]. Furthermore, a reduction in any type of sedentary time is associated with lower health risk in youth. Daily TV viewing in excess of 2 hours is associated with diminished physical and psychosocial health, and a lowered sedentary time reduces body mass index [14]. Physical inactivity has also been associated with an increased likelihood of having several emotional and behavioural problems among boys [15-17]. Hence, an early intervention promoting physical activity and decreasing sedentary behaviour at young age may prevent any future adverse health outcomes.

At present it is not known what the most appropriate method for promoting physical activity among young men is. This would require knowledge and understanding of factors related to deciding to engage in or abstain from physical activity. Interventions to promote physical activity have typically involved teaching individuals the skills to change their activity behaviour, providing knowledge about the goals or opportunities of physical activity, or creating a more physically active environment. In adolescents, multicomponent interventions including school, family and communal elements seem to be most beneficial, yet studies outside the school setting are called for [18,19]. Tailored health communication has proved to be an effective method in promoting healthy behaviours [20,21], but has not been utilized in activation interventions among youth. Innovative approaches taking into account the context of changing youth cultures and trajectories are needed.

In research of behaviour and behaviour change understanding and analysing the phenomena in its socio-cultural setting is essential [22]. Qualitative research methods allow observing the phenomena multifaceted and in the actual context [23]. Thus the socially constructing cultural meanings such as values, attitudes and motives, which direct the actual health behaviour, can be incorporated to the study of the physical activity and activation of young men. Both cultural studies of health and technology are recognised fields of research [24,25], but the combination of these in studies which aims at activation of certain populations through intervention are just emerging.

There is indicative evidence that information and communication technologies (ICTs) such as the Internet and mobile phones can be more effective than other methods for carrying out physical activity interventions among young people [26]. The technology allows a possibility to distribute tailored feedback to a wide range of people and settings in a low cost manner [18,27]. One effective way to convey health information and affect health behaviour could be through games [28,29]. Recent RCT trials show successful interventions [30] and improved health-related behaviour [31,32] through the use of games. Recently, a study showed that using a 3D fantasy game for the treatment of depressive symptoms among adolescents was as effective as conventional counselling and significantly reduced depression, anxiety and hopelessness, and improved quality of life [33]. It seems that the inherent orientation towards playing together transfers from other areas of life into computer games. Social skills are needed, or must be developed, in order to succeed in most of the multi-player games. The social bonding can be so strong that it becomes one of the most important motivating factors for playing the games [34]. This might also apply in the context of physical activation when utilizing a game technology. In this study gamification means the process of increasing user engagement and participation by integrating game mechanics into other youth oriented services, such as communal services, networking and distribution of health information [35].

### Objectives

The overall aim of the study is to provide new evidence-based knowledge for promoting health and wellbeing in young men. The purpose is to set up a multidisciplinary approach for assessing the effectiveness of 1) an interactive, gamified activation method, based on peer networks and participation, on physical activity, aerobic fitness and relationship towards physical activity in young men 2) the gamified activation on physical health, with special emphasis on weight and related factors, and on mental and social health and wellbeing.

The hypotheses are that by the end of the intervention the men in the activation group: 1) are physically more active and fit, and 2) their self-determined and measured health is better and there are fewer obese subjects compared to the control group.

## Methods

### Study design

**Figure 1:**
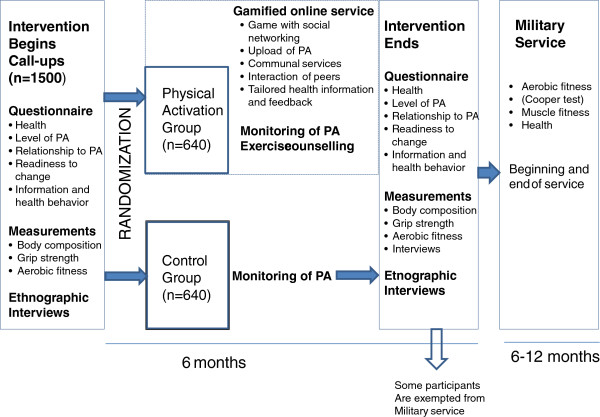
**Study design of the population-based randomized controlled trial.** PA = physical activity.

The study design is a parallel-group randomized controlled trial consisting of an intervention and a control group of young men. The protocol has been registered to the clinical trials register (NCT01376986, ClinicalTrials.gov). The study consists of collection of cross-sectional questionnaire and measurement data at the annual call-ups during the fall 2013 followed by a randomized controlled 6-month physical activity intervention. In addition, the subjects’ entry into military service and its course will be followed (Figure 1).

The statement in favour of the study has been received from the Northern Ostrobothnia Hospital District ethical committee (ETTM123/2009). All the involved organisations have provided permission for conducting the study. All subjects will receive both oral and written information and will be requested for a written consent of their participation. The provided information describes the project and its benefits and possible inconvenience or risks to the subjects, as well as their right to refuse or withdraw from the study without any effects on their future health care or military service. The study is conducted according to Declaration of Helsinki. The subjects’ personal data will be protected using number codes. The collected information will only be used by the research group. The transfer and protection of data will be monitored throughout the project, particularly in interactive web-based and mobile device networks.

### Subjects

In Finland military or civil service is mandatory for all male citizens and annually all 18-year old men are called for service in call-ups. Hence, the entire age cohort attends the call ups except those whose physical or mental health or psychological capacities do not allow independent living. In the call-ups fitness for service is determined based on an interview and a prior medical examination conducted less than six months ago. Conscripts are either assigned or exempted from military service, or ordered to attend a new examination at a later call-up.

The total population of eligible participants include all conscription aged men attending the call-ups in the Oulu area (approximately 1500 annually) in northern Finland.

### Sample size

The primary outcome variable is the change in the time spent in physical activity of different intensities (sedentary, light, moderate and vigorous). The aim is to lower the proportion of inactive men by one third in the intervention group with no change in the control group. At baseline, the estimated number of inactive men is 212 (27.6%, data from the 2009 call-ups). With study power of 80% and significance level of 5%, the calculated sample size is [(27.6 × (100–27.6) + 18.5 × (100–18.5)]/[(27.6-18.5)^2^] × 7.9 = 335 persons/group.

### Setting and randomization

The recruitment is performed at the call-ups for military service in Oulu arranged by the Finnish Defence Forces and City of Oulu every September. Oulu (65°N 25°E) is a growing university town in the northern Finland of approximately 190 000 inhabitants, of which a substantially high proportion are children and adolescents. The unemployment rate of the young people (<25 years) is relatively high in Oulu (19%) compared to other areas in Finland (14%) [36]. Conscription-aged men provide a large, population-based representative sample of young men. All those determined fit for service, who are granted postponement or are exempted from service (n = 1500) will be included in the intervention study. Annually approximately 35% of the conscription aged men in Oulu area are exempted from service, which is the highest proportion in Finland. Adolescents and young men exempted from compulsory military or civil service comprise of a group with a wide range of psychosocial problems and are a target population for supportive interventions [37]. Based on experience for pilot study conducted in 2011, it is estimated that approximately 15% of the men refuse to participate. Thus, approximately 1280 men are expected to participate. These will be randomly allocated into an intervention (n = 640) and control group (n = 640) using computer-generated random numbers. With an estimated 30% dropout rate there will be 447 boys in each group at the end of the intervention.

### The gamified ICT platform for promoting physical activation

The gamified ICT platform forms the basis for different applications. Contrary to previous solutions, subjects do not exercise while playing the game, but their daily activity and exercise is monitored and rewarded in the gamified service. The gamified platform combines several modules: 1) a virtual coach, i.e. avatar for each participant, 2) a game based on participation of peer groups, and achievements supported by rewards from increased physical activity or reduced sedentary behavior 3) possibility for social networking between peers 4) input on physical activity recordings and feedback, 5) communal youth services for supporting coping and an active lifestyle (e.g. through exercise and social counselling), 6) exercise instructions and personal objectives for long-term fitness improvement based on baseline measurements and activity recordings, 7) tailored information content related to health and wellbeing based on a participant's readiness for changing his exercise behaviour. This readiness is evaluated using a modification of Prochaska's Transtheoretical Model of behaviour change [38,39].

The exercise programs provided by professionals will include instructions to physical activity, aerobic and muscle fitness improvement and weight management. Long-term fitness estimation will be provided. The given health information will be evidence-based.

### Intervention

The 6-month intervention will commence at the call-ups (Figure 1). The activation group utilises the abovementioned online gamified activation platform service, whereas the subjects in the control group will continue their normal lives. All subjects will carry a physical activity monitor and the activity data are saved in a database. The control subjects will use blinded monitors which will not provide any feedback for the participants. The questionnaires and measurements will be repeated at the end of the intervention (6 months). The trial protocol and gamified activation service will be pilot tested in 2012 with a smaller sample.

After the intervention the subjects’ entry into military service will be followed up for a period from 18 to 30 months depending on the initiation and length (6–12 months) of service. The course of the service period will be monitored by the Finnish Defence Forces (FDF) which routinely collects data on health and performance of all conscripts attending service. The group assignment will be blinded to the FDF until the end of military service. Examinations measuring aerobic (Cooper - 12 minute running test) [40] and muscle fitness tests [12] are performed when entering service and repeated after its completion. These data will be collected from approximately 70% of the men due to the estimated exemption. This will provide additional information about the impact of the intervention and show the long-term effectiveness of the method aimed at promoting physical activity. It also demonstrates how the activation intervention affects the onset and completion of military service.

### Outcome measures

The questionnaires and physiological measurements have been tested in the annual call-ups in the Oulu area during 2009–2011. Overall, the response rate to the questionnaire has been at or above 62% (2009 N = 770, 73%, 2010: N = 622, 62%, 2011 N = 825, 65%), and 59% or more (59%, 61%, and 72%, respectively) have participated to the measurements.

#### Main and secondary outcome measures

The main outcome measure is self-determined and objectively measured physical activity. Daily physical activity will be followed using a wrist-worn physical activity monitor, Polar Active (Polar Electro Ltd, Finland) [41-46]. This recorder utilizes frequency and regularity of hand movements adjusted for body parameters and a high agreement with energy expenditure has been observed [46]. The device measures physical activity continuously as metabolic equivalents (MET) per 30 s and as time spent at five different zones (very easy, easy, moderate, vigorous, vigorous+), as well as sleep onset and duration. Self-determined physical activity level will be enquired according to questions widely used in health studies [47].

The type of physical activity (sitting/standing, walking, running, cycling and driving) will be tracked using a smartphone with triaxial accelerometers [48-50]. The recognition is based on different features of the acceleration signal and classification is done using *knn* (k nearest neighbours) and QDA (quadratic discriminant analysis) classifiers. The software has a real-time recognition accuracy of 95% when the phone is in the pocket of the subject’s trousers [50].

The secondary outcome is the proportion of overweight and obese boys.

#### Questionnaires

The following cross-sectional questionnaire data will be collected during the military call-ups: 1) health survey consisting of questions related to health behaviour, diet, sleep, psychosocial and socioeconomic factors, quality of life, depressive symptoms (Raitasalo’s modification of the short form of the Beck Depression Inventory [51,52], and substance abuse; 2) amount of physical activity [47] and sitting time, self-rated physical fitness, 3) relationship to physical activity: a questionnaire focusing on physical activity or lack thereof, and the underlying motives, 4) information behaviour and practices in relation to the stages of change of the transtheoretical model [38,39], the use of media, games and technology.

#### Ethnographic and interview data

Participant observation and subject interviews are conducted in several phases. Firstly, the objective is to understand specific values, motivations and cultural meanings in general in youth culture which affect adolescent’s health behaviour [53]. Secondly, an in-depth description is constructed based on their experiences and opinions of the intervention in order to improve the methods further [54,55]. In this way the ethnographic work also provides feedback between the target group and service developers.

#### Measurements

Height will be measured with a ruler. Waist circumference will be measured midway between the lowest rib and the iliac crest. Body composition (fat mass, fat free mass, percentage body fat), BMI and weight will be assessed by bioelectrical impedance assessments (DSM-BIA; direct segmental multi-frequency bioelectrical impedance analysis). For this purpose InBody 720 Body composition analysis (Biospace Co., Ltd., Seoul, Korea) will be used. The accuracy of measurement is high in comparison to e.g. the dual-energy x-ray absorptiometry (DXA) method [56] and practical in large epidemiological studies with limited time for examining each subject [11]. Bilateral grip strength of both hands will be measured using a dynamometer (Saehan, SAEHAN Corporation, Korea). During the examination, the subjects stand with their legs apart and elbow at 90° angle, and grip the instrument with maximum strength.

Aerobic fitness will be evaluated using a fitness test (Polar Fitness Test™, Polar Electro, Finland) conducted while resting comfortably during ca. 5 minutes. The Polar Fitness Test™ predicts maximal oxygen uptake (ml/min/kg) from the resting heart rate, heart rate variability, gender, age, height, body weight and self-assessed physical activity [57]. Heart rate variability (R-R intervals) will be measured from 255 heart beats (3–5 min) with a sampling frequency of 1000 Hz [58]. During the military service, all conscripts undergo muscle fitness tests [12] performed twice by the FDF. Aerobic fitness with Cooper’s 12-minute running test [40] will be performed at the beginning and end of the military service.

### Statistical analysis

Descriptive analyses will be performed utilising the results of the questionnaires and measurements. Absolute and percentage changes from baseline will be calculated for continuous variables. The paired samples t test will be used to compare means of the within group change from baseline, and the t test for independent samples will be used to compare means between the treatment and the control group. The analysis of variance for repeated measures will be used to analyse the intra-group-by-time-effect and the group-by-time interaction and to adjust for possible confounding factors over the study period. The changes in activity and weight during the trial and follow-up will also be examined by multiple linear regression analyses with generalized estimation equations (GEE) to account for correlations between repeated measurements. Association between the response and explanatory factors in the aggregated data will first be analysed using cross tabulation and the Pearson product–moment correlations. Multiple linear and logistic regression analysis using all variables associated with activity level and weight in univariate analyses will be performed to evaluate the determinants of change within the exercise group and the pooled groups. Data will be analysed using both intention to treat and per protocol basis.

## Discussion

The results of this study will provide evidence on the effectiveness of the online gamified activation service for increasing the activity and reducing inactivity in young men. This is the first study to combine a gamified service with objective continuous measurement of physical activity in daily life; i.e. also when the subject is not using the service. The study also provides novel information on the health status, physical fitness, motivational factors and profiles of physically active and inactive young men. This information can be utilized in future development of tailored gamified services. Promotion of physical activity at young age provides an early intervention for preventing any future adverse health effects. Hence, the produced physical activation method has broad public health significance.

The developed new model of gamification used for promoting physical activity will provide a new approach for reaching young people. Tailored health messages are delivered based on applying the transtheoretical model that is augmented with the concept of information behaviour to identify the participants’ stages of exercise behaviour change. This study will explore new possibilities to direct the health behaviour of young men without many times ineffective and provoking top-down coercive health instructing, which may have opposite effects that was intended among adolescents [59]. For finding new, affecting, and at same time entertaining and socially acceptable means for adolescents, their active participation to the development process is essential. The produced gamified platform may exert other positive social effects which may directly or indirectly improve health and wellbeing.

There are challenges in this trial. Firstly, the young men form a challenging target group that has not been widely studied. Furthermore, the most inactive young men may not be willing to participate to the study resulting in selection bias. Based on our previous pilot study, this will not likely affect the main results since the main characteristics of the participants and non-participants did not differ. Secondly, in previous studies compliance to wear physical activity monitors has been low. Although the Internet is a promising medium to deliver behaviour change programs, previous research have shown low exposure rates to web-based interventions [60]. The increased use of smartphones may change this due improved access to internet from almost anywhere. As a strength of our study, the target group is actively involved in the design of the gamified activation service and its social elements to provide appealing contents. Online social features have reduced attrition from health behaviour change interventions, especially in populations with low social support [61]. Tailored health messages will be delivered based on an application of the transtheoretical model. The degree of message tailoring may influence engagement to the intervention program, which is associated with positive health behaviour change [62].

In conclusion, the planned multidisciplinary co-operation aims to produce information of health and factors affecting health behavior in young men. This information will be utilized for the development of new methods for physical activation of young people. The development work is conducted in co-operation with the participants and utilizing important elements of youth culture. If successful, this unique intervention to activate young men can provide a translational model for community use. Also, if proven effective, the produced physical activation method can be utilized as such or tailored to other selected populations or age groups.

## Abbreviations

BMI: Body mass index; DSM-BIA: Direct segmental multi-frequency bioelectrical impedance analysis; DXA: Dual x-ray absorptiometry; FDF: Finnish Defence Forces; GEE: General estimation equation; ICT: Information and communication technology, knn, k nearest neighbours; MET: Metabolic equivalent; QDA: Quadratic discriminant analysis; RCT: Randomised controlled trial.

## Competing interests

The authors declared that they have no competing interests.

## Authors’ contributions

All authors have been involved in the planning of the study. All authors contributed to developing the protocols and intervention materials. RA drafted the manuscript and all authors were involved in revisiting it for intellectual content. All authors have revised the manuscript and read and accepted the final version.

## Pre-publication history

The pre-publication history for this paper can be accessed here:

http://www.biomedcentral.com/1471-2458/13/32/prepub
